# The Relationship Between Social Anxiety and Depression Among Rural High School Adolescents: The Mediating Role of Social Comparison and Social Support

**DOI:** 10.3390/healthcare13050533

**Published:** 2025-02-28

**Authors:** Yang Cao, Jia Wang, Ziqin Huang, Yiming Qin, Siyu Gao, Huiping Zhang, Meng Yuan, Xinfeng Tang

**Affiliations:** 1 Department of Sociology, Zhejiang University, Hangzhou 310058, China; 11922018@zju.edu.cn; 2 Department of Psychology, Renmin University of China, Beijing 100872, China; jiawang@niglas.ac.cn (J.W.); ziqinhuang@ruc.edu.cn (Z.H.); 201922240049@mail.bnu.edu.cn (Y.Q.); 3 Institute of Social Work and Social Policy, China University of Political Science and Law, Beijing 100088, China; gaosiyu@connect.hku.hk; 4 Center for Studies of Sociological Theory and Method, Renmin University of China, Beijing 100872, China; zhp0205@ruc.edu.cn; 5 Department of Social Work, Renmin University of China, Beijing 100872, China; 6 Mental Health Education and Counseling Center, Renmin University of China, Beijing 100872, China

**Keywords:** depression, social anxiety, social comparison, social support, high school students

## Abstract

**Objective**: This study aimed to explore the impact of social anxiety on depression among high school students and examine the parallel mediating roles of social comparison and social support. **Methods**: A total of 806 rural high school students were surveyed using the Short Mood and Feelings Questionnaire, the Social Phobia Inventory, the Chinese version of the Social Comparison Orientation Scale, and the Multidimensional Scale of Perceived Social Support. **Results**: Social anxiety and social comparison were significantly and positively correlated with depression, while social support exhibited a significant negative correlation with depression. The direct effect of social anxiety on depression was also found to be significant. Moreover, social comparison and social support both played significant parallel mediating roles in the relationship between social anxiety and depression. **Conclusions**: Social anxiety has a direct positive predictive effect on depression, and this effect can be indirectly mediated through the parallel roles of social comparison and social support.

## 1. Introduction

Depression is a globally prevalent mental health condition [1]. Individuals suffering from depression often feel hopelessness, lack energy, and in severe cases, may even engage in self-harm or suicidal behavior [2,3]. Depression in adults often begins during adolescence [4]. According to the 2022 World Mental Health Report published by the World Health Organization, 14% of adolescents worldwide suffer from mental health disorders [5], with a rapidly increasing trend [6]. High school students are particularly prone to psychological health issues due to the immense pressure of college entrance exams [7]. Between 2010 and 2020, the depression detection rate among high school students in mainland China was 28% [8]. Some researchers have observed that the prevalence rate of depression among middle school students in rural areas is higher than that in urban areas [9]. Furthermore, in rural areas, adolescents from lower socioeconomic backgrounds, especially females, are at greater risk of experiencing depression [10,11]. Depression is closely linked to negative outcomes such as academic decline, impaired social functioning, and suicidal behaviors [12]. Consequently, investigating the causes and influencing factors of depression among rural high school students is of paramount importance for the future development of adolescent mental health.

### 1.1. Social Anxiety and Depression Among High School Students

A substantial amount of research has explored the risk factors for depression, with interpersonal relationships and perceptions of these relationships being significant contributors [13,14]. Specifically, social anxiety has been closely linked to adolescent depression in many countries. Social anxiety refers to excessive tension, worry, and fear in social situations, with an intensity that is out of proportion to the actual social context [15]. According to a 2017 large-scale mental health survey of adult populations in 26 countries with high-, middle-, and low-income levels, the 30-day, 12-month, and lifetime prevalence rates of social anxiety disorder are 1.3%, 2.4%, and 4.0%, respectively [16].

Numerous cross-sectional studies have demonstrated a significant association between social anxiety and depression, with individuals exhibiting higher levels of social anxiety being more likely to develop depressive symptoms [17,18,19]. Other cross-sectional research has suggested that social anxiety and depression are highly comorbid [20,21], with social anxiety frequently occurring both before and after the onset of depression [22]. Longitudinal studies have found that social anxiety can act as a significant predictor of subsequent depression. For instance, a study of three adolescent samples (Sample 1 = 1116 adolescents, Sample 2 = 1423 adolescents, and Sample 3 = 549 adolescents) across three measurement points over a one-year interval revealed that social anxiety symptoms are crucial in triggering depressive symptoms [23]. Another longitudinal study involving 501 adolescents at three time points found that social anxiety precedes and is a strong predictor of depressive symptoms, while depression does not predict social anxiety, which indicates the absence of a bidirectional relationship. Moreover, social anxiety has been found to positively predict depression [24]. Lastly, a two-year study involving 246 pairs of community adolescents and their biological mothers from the US confirmed that social anxiety positively predicts depression, while depression fails to predict future changes in social anxiety [25].

In addition to the aforementioned direct effects, social anxiety has an indirect effect on depression through mediating variables. For example, in cross-sectional studies, alexithymia, loneliness, and regulatory emotional self-efficacy have been found to partially mediate the association between social anxiety and depression [26,27,28]. Furthermore, poor sleep quality [29] and eating disorders [30] have been identified as mediators in the link between social anxiety and depression. Longitudinal studies indicate that anxiety can positively predict depression, with avoidance acting as a mediator [31], and social anxiety precedes depressive symptoms, with self-esteem [32], low sociability, interpersonal oversensitivity, and social chronic stress serving as mediators [33]. Evidently, the mediating variables in the relationship between social anxiety and depression predominantly involve individual factors, and the underlying mechanisms of their relationship warrant further exploration.

### 1.2. The Relationship Between Social Anxiety, Social Comparison, and Depression

In the development of depression, factors related to interpersonal and social interactions are indispensable. For example, research has shown that the process of social comparison plays a crucial role in the etiology and maintenance of depression [34,35]. Social comparison refers to the process by which individuals assess themselves in relation to others, typically resorting to comparisons when they are unable to evaluate themselves objectively or directly. In such instances, individuals often use other people as reference points for self-assessment [36]. The content of these comparisons encompasses various psychological aspects, including behavior, emotions, and cognition [37].

Social hierarchy theory posits that negative social comparisons are likely to make individuals perceive themselves as inferior to others, thereby increasing their depression risk [38,39]. Social comparison has been shown to significantly and positively predict depression [40,41,42]. For instance, a depression survey conducted across 23 countries found that nations with higher levels of income inequality have a higher prevalence of depressive symptoms, with income inequality acting as a source of stress through social comparison and exerting a negative influence on depression [43]. A longitudinal analysis by Osafo Hounkpatin et al. examined data from Wisconsin residents in 1992 and 2003, as well as UK residents in 2002 and 2008, to investigate the relationship between income and depression, finding that the social comparison of income was a stronger predictor of depressive symptoms [44]. Therefore, individuals with a higher tendency for negative social comparison are more likely to experience negative emotions, perceiving themselves as inferior to others in social situations, thus elevating their depression levels.

Similarly, social anxiety is a significant predictor of social comparison. Research has demonstrated a positive correlation between social comparison and social anxiety, with higher levels of social anxiety being associated with a stronger tendency for social comparison [45,46]. Individuals with elevated social anxiety exhibit memory biases, are more sensitive to negative feedback, and persistently engage in negative self-evaluation [47], which often leads to negative social comparisons [48,49]. Therefore, this study proposes the following hypothesis:

**Hypothesis** **1.** *Social comparison mediates the relationship between social anxiety and depression*.

### 1.3. Social Anxiety, Social Support, and Depression

Among the numerous factors influencing adolescent depression, in many countries, social support has been strongly associated with depression. Social support refers to the assistance that individuals perceive from family, friends, and others. It manifests in the form of social ties, social networks, and interpersonal friendships, representing stable interpersonal relationships [50]. Social support encompasses three primary types: tangible, emotional, and informational support [51].

The interpersonal relationship model of depression posits that individuals with depression frequently seek reassurance from others, which exacerbates their distress. When external support wanes or comfort is rejected, this distress intensifies, thereby increasing their vulnerability to depression [52]. Research has shown that social support negatively predicts depression. Individuals with low social support are more prone to depressive symptoms [53,54,55]. A study by Höltge et al. tracking three waves of data over three years found that support from family and friends was significantly negatively associated with depression [56]. Scholars have also discovered that, when measuring adolescent social support and depressive symptoms every six weeks over a six-month period, insufficient support from parents and classmates, as opposed to insufficient peer support, was more influential in adolescent depression. These findings highlight the role of social support in reducing depressive symptoms [57]. A longitudinal study in Finland, spanning from adolescence to adulthood, revealed that reduced levels of social support are associated with increased depressive symptoms [58]. Consequently, individuals with lower social support are more prone to elevated depressive symptoms.

On the other hand, social anxiety has been found to negatively predict social support [59,60]. A study collecting data from adolescents in the fall of sixth grade and the spring of seventh grade revealed that increased social anxiety is associated with reduced peer support and impairs the ability to effectively engage in peer interactions [61]. A study by Gallagher et al. [62], which conducted follow-ups with adolescents hospitalized for psychiatric conditions over 9 months and 18 months, confirmed that social anxiety significantly negatively predicts social support. Consequently, this study proposes the following hypothesis:

**Hypothesis** **2.** *Social support mediates the relationship between social anxiety and depression*.

### 1.4. Current Research

The existing literature indicates that current research on the relationship between social anxiety and depression reveals several limitations. First, the mediating variables between social anxiety and depression primarily focus on individual factors, while the influence of interpersonal and social interaction factors remains underexplored. Second, there is a noticeable lack of studies that simultaneously examine the mediating roles of social comparison and social support in the relationship between social anxiety and depression.

**Figure 1:**
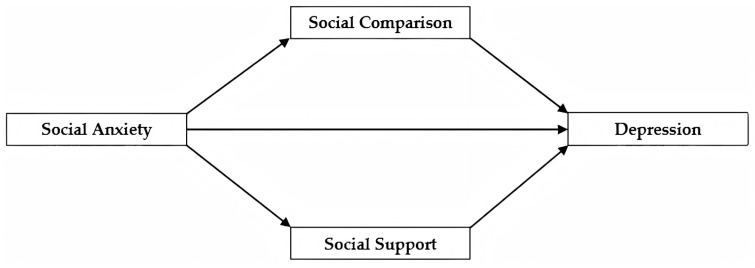
Hypothetical model.

Therefore, this study aimed to explore the impact of social anxiety on depression among high school students and examine the parallel mediating roles of social comparison and social support. To more comprehensively examine the underlying mechanisms of the social anxiety–depression relationship, this study adopted a survey method to investigate high school students in rural areas of mainland China. Specifically, this study took into account the predictive roles of social anxiety, social comparison, and social support in depression, and a parallel mediation model (Figure 1) was proposed to test the aforementioned hypotheses (Hypothesis 1: Social comparison mediates the relationship between social anxiety and depression. Hypothesis 2: Social support mediates the relationship between social anxiety and depression).

## 2. Materials and Methods

### 2.1. Study Participants

This study employed a convenience sampling method to conduct a questionnaire survey with 806 students from a high school in a county of Huai’an, Jiangsu Province, China (Table 1). Participants anonymously completed the survey on site; their anonymity was ensured to protect their privacy, and their participation was entirely voluntary, with the option to withdraw at any time. Based on the criterion that missing and implausible data should account for no more than 10% of the total responses for each scale item, 760 valid questionnaires were collected, resulting in a valid response rate of 94.29%. Among the respondents, 48.8% identified as female, 48.7% identified as male, and the average age was 16.43 years. Of the valid responses, 46.7% of participants scored ≥19 on the Social Phobia Inventory (SPIN), with a score of 19 serving as the threshold for identifying significant social anxiety [63].

### 2.2. Instruments

#### 2.2.1. The Short Mood and Feelings Questionnaire

The Short Mood and Feelings Questionnaire (SMFQ), developed by Angold et al. [64], comprises 13 items. Cheng et al. [65] validated the reliability and validity, demonstrating that it is an effective tool for screening depression levels in Chinese children and adolescents. The scale uses a 1–3 Likert scoring system (ranging from 0 = “None” to 2 = “Frequent”), with higher total scores indicating higher levels of depression. In this study, the Cronbach’s alpha coefficient for the SMFQ was 0.894.

#### 2.2.2. The Social Phobia Inventory

The Social Phobia Inventory (SPIN), developed by Connor et al. [66] and revised by Xiao et al. [63]. Its reliability and validity were verified by scholars for application within the Chinese rural adolescent population [67]. The scale consists of 17 items across three dimensions: fear, avoidance, and physiological symptoms. Scoring is based on a 5-point Likert scale ranging from 0 to 4 (0 = “None”, 1 = “A little”, 2 = “Some”, 3 = “Very”, and 4 = “Extremely”), with a total score range from 0 to 68. The higher the score, the greater the severity of the social anxiety, with a score exceeding 19 suggesting the presence of social anxiety. In this study, the Cronbach’s alpha coefficient for the SPIN was 0.934.

#### 2.2.3. The Social Comparison Orientation Scale

The Social Comparison Orientation Scale was developed by Gibbons and Buunk [68] and revised for the Chinese version by Wang et al. [69]. Its reliability and validity were verified by scholars for application within the Chinese rural adolescent population [70]. The scale consists of 11 items and utilizes a 5-point Likert scale for scoring: 1 = “Strongly Disagree”, 2 = “Somewhat Disagree”, 3 = “Unsure”, 4 = “Somewhat Agree”, and 5 = “Strongly Agree”. The maximum total score is 55. A higher score indicates a stronger orientation for social comparison, with individuals more likely to relate events happening to others to themselves and to show a greater interest in the thoughts and behaviors of people in similar circumstances. In this study, the Cronbach’s alpha coefficient for the Social Comparison Orientation Scale was 0.967.

#### 2.2.4. The Multidimensional Scale of Perceived Social Support

The Multidimensional Scale of Perceived Social Support (MSPSS) was developed by Zimet et al. [71], and its reliability and validity were verified by Yang et al. [72] for application within the Chinese secondary school population. The Chinese version consists of 12 items across 3 dimensions and uses a 4-point Likert scale: 1 = “Strongly Disagree”, 2 = “Disagree”, 3 = “Agree”, and 4 = “Strongly Agree”. A higher total score indicates a higher level of perceived social support. In this study, the Cronbach’s alpha coefficient for the MSPSS was 0.968.

### 2.3. Procedure

This study employed a random sampling method, selecting high school students from a county in Huai’an City, Jiangsu Province, to conduct a questionnaire survey. The survey was conducted during class meetings. After signing an informed consent form, participants filled out the questionnaires on site. This study was approved by the Institutional Review Board of the Department of Psychology of Renmin University of China. A teacher was assigned to each class to distribute the questionnaires. Prior to the distribution, students were informed of the guidelines for completing the questionnaire and were told that its purpose was to gain a better understanding of their mental health status. Confidentiality was ensured, and an anonymous data collection system was implemented to protect students’ privacy. Participation was voluntary, with participants having the right to refuse or withdraw at any time. Once the questionnaires were collected, the data were entered into SPSS Statistics 26.0 and analyzed.

### 2.4. Data Analysis

The data were analyzed in SPSS using tests for common method bias, descriptive statistics, regression, and correlation analysis. The mediation effects were tested using Mplus 8.10.

## 3. Results

### 3.1. Test for Common Method Bias

This study employed Harman’s single-factor test to assess common method bias. The results indicated that 11 factors had eigenvalues greater than 1, with the percentage of variance explained by the first common factor being 23.65%, which is below the critical threshold of 40%, and revealed that there was no significant common method bias in this study.

### 3.2. Descriptive Statistics and Correlation Analysis of Study Variables

Descriptive statistics and Pearson correlation analysis were performed on social anxiety, social comparison, social support, and depression (Table 2). The results of the correlation analysis revealed a significant positive correlation between social anxiety and depression (r = 0.513, *p* < 0.01). Furthermore, significant relationships were observed among the primary variables, providing a foundation for testing the parallel mediation model.

### 3.3. Regression and Mediation Effect Analysis

This study employed hierarchical regression. In Model 1, gender, maternal education level, paternal education level, and living standard were controlled as variables, with depression serving as the dependent variable. In Model 2, social anxiety was introduced as the independent variable. In Model 3, social comparison and social support were included as mediating variables. Hierarchical regression analysis was conducted using SPSS, with the results presented in Table 3.

As shown in Table 3, in Model 1, R^2^ = 0.051, adjusted R^2^ = 0.045, F = 8.172, *p* < 0.001, indicating that gender and living standard exert a significant positive influence on depression. In Model 2, R^2^ = 0.282, adjusted R^2^ = 0.276, F = 47.513, *p* < 0.001, suggesting that both gender and social anxiety have a significant positive effect on depression. In Model 3, R^2^ = 0.373, adjusted R^2^ = 0.366, F = 51.324, *p* < 0.001, demonstrating that gender, social anxiety, and social comparison all significantly positively predict depression, while social support had a significant negative impact on depression.

**Figure 2:**
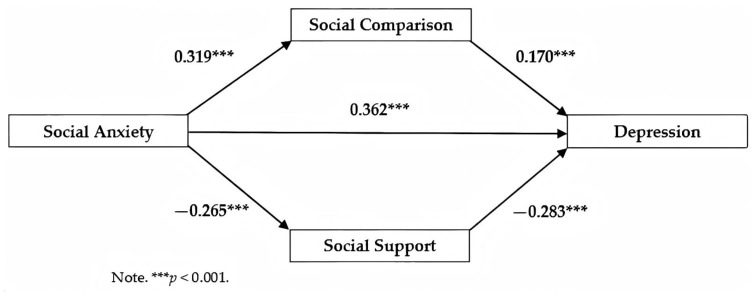
The relationship between depression and social anxiety: the mediating roles of social comparison and social support.

Subsequently, this study employed social anxiety as the independent variable, and depression as the dependent variable, with social comparison and social support as mediating variables, and gender as a control variable. Path analysis was conducted using Mplus, with Maximum Likelihood (ML) estimation, and the standardized results are shown in Figure 2 and Table 4.

Bootstrap sampling with 10,000 resamples was employed to examine whether the total, indirect total, indirect, and direct effects among the four variables—social anxiety, social comparison, social support, and depression—were statistically significant. The analysis of the relationship between depression and social anxiety, along with the mediating effects of social comparison and social support, is presented in Table 5.

In summary, social anxiety exerted both a direct effect on depression and an indirect effect through the mediation of social comparison and social support. The direct effect accounted for 73.73% of the variance, while the indirect effect accounted for 26.27%, with the direct effect carrying a greater weight than the indirect effect. In terms of the indirect effect, the weights of social comparison and social support were relatively similar.

Firstly, the total predictive effect of social anxiety on depression was significant, with an estimated value of 0.491 and a 95% confidence interval (CI) of [0.408, 0.565]. The direct effect of social anxiety on depression was also significant, with a path coefficient of β = 0.362, *p* < 0.001, and a 95% CI of [0.270, 0.447].

Secondly, the path from social anxiety to social comparison was significant, with a path coefficient of β = 0.319, *p* < 0.001, and a 95% CI of [0.243, 0.388]. The path from social comparison to depression was also significant, with a path coefficient of β = 0.170, *p* < 0.001, and a 95% CI of [0.105, 0.229]. Social anxiety exerted an indirect effect on depression through social comparison, with a significant indirect effect weight of 11.00%, an estimated value of 0.054, *p* < 0.001, and a 95% CI of [0.031, 0.080]. These findings support Hypothesis 1.

Lastly, the path from social anxiety to social support is significant, with a path coefficient of β = −0.265, *p* < 0.001, and a 95% CI of [−0.347, −0.174]. The path from social support to depression is also significant, with a path coefficient of β = −0.283, *p* < 0.001, and a 95% CI of [−0.352, −0.213]. Social anxiety influences depression through social support, with a significant indirect effect, accounting for 15.27% of the total effect. The estimated value of the indirect effect is 0.075, *p* < 0.001, with a 95% CI of [0.049, 0.108]. These findings support Hypothesis 2.

Hence, it can be inferred that social comparison and social support play a significant parallel mediating role between social anxiety and depression.

## 4. Discussion

Using a questionnaire survey, this study investigated the relationships among depression, social anxiety, social comparison, and social support, with depression as the outcome variable and the latter three variables as predictors. The results indicate that social anxiety serves as a predictor of depression, while social comparison and social support function as parallel mediators between social anxiety and depression.

### 4.1. The Mediating Role of Social Comparison

The results of this study reveal that social comparison plays a significant mediating role between social anxiety and depression, suggesting that individuals with social anxiety may exacerbate their depressive symptoms through frequent negative social comparisons. This finding aligns with social comparison theory [36], which posits that individuals tend to assess their opinions and abilities by comparing themselves to others. In the context of social anxiety, individuals often exhibit a negative bias, overestimating the strengths of others while underestimating their own social abilities, which thereby leads to heightened negative social comparisons [73,74,75]. This cognitive pattern may reinforce an individual’s negative self-assessment of their social competence, thus intensifying the onset of depression [76,77,78,79,80]. An alternative explanation is that individuals with higher levels of social anxiety are more prone to re-evaluating social relationships through a hierarchical lens, perceiving themselves as inferior and subject to others’ disdain [81] and so are more likely to develop materialistic thoughts [82]. Consequently, they tend to report more negative evaluations during social comparisons. Therefore, individuals who engage in more social comparisons are more likely to seek to reduce the disparity between themselves and others, which, in turn, increases their susceptibility to depression.

Furthermore, the mediating role of negative social comparison is likely to exhibit cultural specificity. In collectivist cultures, individuals are highly sensitive to social norms and the evaluations of others, and the effects of negative social comparison are likely to be more pronounced [83,84,85]. In contrast, in individualistic cultures, individuals tend to emphasize self-directed evaluations of competence [86], thereby attenuating the impact of negative social comparisons. Although this study measured the mediating role of social comparison within a collectivist culture, the meaning of social comparison differs between collectivist and individualist cultures. In a collectivist culture, social comparison may be more concerned with whether one meets societal expectations and fears negative evaluation. In contrast, in an individualist culture, social comparison is more likely to be based on personal standards of self-assessment, rather than excessive reliance on external comparisons. Thus, while both cultures examine social comparison, the underlying mechanisms of this process may vary if we attempt to generalize our findings to an individualist cultural context. Therefore, the mechanisms underlying the role of social comparison as a common variable in cross-cultural research may differ. These cultural differences provide valuable avenues for future research to further explore this phenomenon.

### 4.2. The Mediating Role of Social Support

This study reveals that social support serves as a significant mediator between social anxiety and depression. Individuals with social anxiety often reduce their social interactions due to the fear of negative evaluation or social embarrassment, which, in turn, limits their opportunities to receive social support [60]. However, for those who can maintain a certain level of social support, their emotional regulation abilities and access to resources are enhanced, thereby reducing their risk of depression [87]. The significant mediating effect of social support between social anxiety and depression can be further elucidated through the lens of social capital theory. This theory emphasizes that resources obtained through social networks, such as information [88,89], emotional support [90], and practical assistance [91,92], form the core components of an individual’s social capital. For individuals with social anxiety, their tendency to reduce social interactions may undermine their ability to acquire and maintain social capital, which is a key factor that contributes to depression [93,94,95].

It is noteworthy that the impact of social support may be influenced by both the individual’s perception and the type of support provided. For instance, perceived social support may be more crucial than actual social support in alleviating the negative effects of depression [96]. Furthermore, among perceived social support, the effects of family support and friendship support on depression may vary across different adolescent developmental stages [97]. Future research could further explore the mechanisms underlying the different types of social support. Increasing social support for individuals with social anxiety is not only a key objective of psychological interventions but also provides a practical direction for depression prevention.

### 4.3. Limitations

This study has several limitations. Firstly, participants were recruited from a rural high school in Huai’an, Jiangsu Province, which may limit the generalizability of the findings. Future research could employ cross-regional random sampling to survey lower-grade students from rural high schools of varying sizes, thereby providing a more comprehensive understanding of the issues surrounding depression. In addition, our research focused on a community sample rather than a clinical sample, and therefore, our conclusions may not be directly generalizable to clinical populations. Secondly, this study utilized a single questionnaire method, which constrained the depth of the data. Future investigations might consider adopting experimental approaches to more thoroughly explore the relationship between social anxiety and depression. Furthermore, this study relied on self-reported measures, which may be prone to recall bias; the inclusion of clinical diagnostic tools could offer a more comprehensive evaluation. Thirdly, the cross-sectional design employed in this study limits the ability to ascertain causal relationships between social anxiety, social comparison, social support, and depression. Hence, subsequent research could prioritize longitudinal designs to provide causal evidence for the parallel mediation model. Fourthly, the significant direct effect between social anxiety and depression suggests the presence of unexplored mediating mechanisms; this is an interesting avenue for future research. Lastly, this study employed a manifest variable approach for analysis, rather than a latent variable model, which may result in imprecise estimations of the relationships between variables [98]. Future research could consider utilizing a latent structural equation model to correct measurement error and enhance the robustness of the findings.

## 5. Conclusions

In summary, this study established a parallel mediation model to examine the impact of social anxiety, social comparison, and social support on depression in high school students. The findings demonstrated a significant positive correlation between social anxiety and depression. Furthermore, both social comparison and social support served as independent mediating factors in the relationship between social anxiety and depression. These results provide an empirical foundation for designing interventions to alleviate depression in high school students. This research particularly underscores the importance of addressing social anxiety, reducing tendencies for negative social comparison, and enhancing social support as key strategies for mitigating depression; thus, it holds substantial implications for the development of effective interventions for adolescent depression. This is reflected in three levels: Firstly, at the family level, it is essential to implement parent training to enhance parental support. Specifically, during the parenting process, parents should foster opportunities for children to engage in social interactions and cultivate greater self-confidence. Parents should serve as social role models, creating a supportive environment for the child and minimizing comparisons with others. Secondly, at the school level, it is important to encourage students’ social interactions and reduce reliance on academic performance comparisons. Schools should provide more support from peers and teachers. Thirdly, at the individual level, it is crucial to screen for social anxiety in individuals with depression. Those with both social anxiety and depression should receive timely interventions for social anxiety. Additionally, individuals with social anxiety and depression should be encouraged to minimize social comparisons and seek more social support. Based on our findings, we offer two recommendations for future research. First, further investigation is needed into other mediating factors influencing social anxiety and depression among rural adolescents, such as negative self-perception, poor interpersonal relationships, and social exclusion. Second, future studies could consider designing longitudinal models to examine the causal relationships between variables and their changes over time.

## Figures and Tables

**Table 1:** Respondents’ demographic information.

Statistical Indicators	Categories	Number of Participants (n)	Percentage (%)
Gender	Male	370	48.7
	Female	371	48.8
	Missing	19	2.5
Age	15–18 years (inclusive)	749	98.6
	19 years	2	0.2
	Missing	9	1.2
Maternal Education Level	Junior High School or Below	595	78.3
	High School, Vocational School, or Technical School	139	18.3
	College (Associate or Bachelor’s Degree)	15	2.0
	Master’s Degree or Above	2	0.3
	Missing	9	1.1
Paternal Education Level	Junior High School or Below	464	61.1
	High School, Vocational School, or Technical School	251	33.0
	College (Associate or Bachelor’s Degree)	35	4.6
	Master’s Degree or Above	2	0.3
	Missing	8	1.0
Living Standard	Poor	55	7.2
	Modestly Affluent	166	21.8
	Average	507	66.7
	Relatively Affluent	25	3.3
	Very Affluent	3	0.4
	Missing	4	0.6

**Table 2:** Correlations and descriptive statistics for each variable.

		M	SD	1	2	3	4
1	Social Anxiety	8.23	5.62	1			
2	Social Comparison	19.21	13.00	0.367 **	1		
3	Social Support	33.83	7.57	−0.240 **	−0.096 *	1	
4	Depression	34.51	8.24	0.513 **	0.343 **	−0.371 **	1

Note. M: mean, SD: standard deviation. ** *p* < 0.01,* *p* < 0.05.

**Table 3:** Regression analysis.

Variable	Model 1		Model 2		Model 3	
	*β*	*t*	*β*	*t*	*β*	*t*
Gender	0.200	5.052 ***	0.093	2.637 **	0.110	3.333 ***
Maternal Education Level	0.008	0.192	0.046	1.232	0.030	0.865
Paternal Education Level	0.045	1.055	0.072	1.918	0.066	1.878
Living Standard	−0.100	−2.518 *	−0.051	−1.472	−0.025	−0.759
Social Anxiety			0.498	13.945 ***	0.367	9.975 ***
Social Comparison					0.177	5.106 ***
Social Support					−0.262	−7.827 ***
R^2^ (∆R^2^)	0.051		0.282 (0.231)		0.373 (0.091)	
Adjusted R²	0.045		0.276		0.366	
*F*	8.172 ***		47.513 ***		51.324 ***	

Note. *** *p* < 0.001,** *p* < 0.01,* *p* < 0.05.

**Table 4:** Path coefficients.

Path	Estimate	95% Confidence Interval	Significance *p*
Social Anxiety → Depression	0.362	[0.270, 0.447]	<0.001
Social Anxiety → Social Comparison	0.319	[0.243, 0.388]	<0.001
Social Comparison → Depression	0.170	[0.105, 0.229]	<0.001
Social Anxiety → Social Support	−0.265	[−0.347, −0.174]	<0.001
Social Support → Depression	−0.283	[−0.352, −0.213]	<0.001
Social Anxiety → Social Comparison → Depression	0.054	[0.031, 0.080]	<0.001
Social Anxiety → Social Support → Depression	0.075	[0.049, 0.108]	<0.001

**Table 5:** Mediating effect analysis.

Path	Estimate	95% Confidence Interval	Proportion of Total Effect
Total Effect	0.491	[0.408, 0.565]	
Direct Effect (Social Anxiety → Depression)	0.362	[0.270, 0.447]	73.73%
Total Indirect Effect	0.129	[0.096, 0.167]	26.27%
Social Anxiety → Social Comparison → Depression	0.054	[0.031, 0.080]	11.00%
Social Anxiety → Social Support → Depression	0.075	[0.049, 0.108]	15.27%

## Data Availability

The raw data supporting the conclusions of this article will be made available by the authors on request.

## References

[1] Herrman H., Patel V., Kieling C., Berk M., Buchweitz C., Cuijpers P., Furukawa T.A., Kessler R.C., Kohrt B.A., Maj M. (2022). Time for united action on depression: A Lancet-World Psychiatric Association Commission. Lancet.

[2] Jensen M.M. (2005). Introduction to Emotional and Behavioral Disorders: Recognizing and Managing Problems in the Classroom.

[3] Harrington R., Fudge H., Rutter M., Pickles A., Hill J. (1990). Adult outcomes of childhood and adolescent depression. I. Psychiatric status. Arch. Gen. Psychiatry.

[4] Hong H. (2005). Family group counseling on depression and behavioral problems in children and teenagers. Chin. J. Clin. Rehabil..

[5] Freeman M. (2022). The World Mental Health Report: Transforming mental health for all. World Psychiatry.

[6] Barker M.M., Beresford B., Bland M., Fraser L.K. (2019). Prevalence and Incidence of Anxiety and Depression Among Children, Adolescents, and Young Adults With Life-Limiting Conditions: A Systematic Review and Meta-analysis. JAMA Pediatr..

[7] Tian Y., He M. (2021). Mediating effect of self-compassion and experiential avoidance between mindfulness and mental health of high school students in Tianjin city. Med. Soc..

[8] Yu X., Zhang Y., Yu G. (2022). Prevalence of mental health problems among senior high school students in mainland of China from 2010 to 2020: A meta-analysis. Adv. Psychol. Sci..

[9] Liu F., Song X., Shang X., Wu M., Sui M., Dong Y., Liu X. (2020). A meta-analysis of detection rate of depression symptoms among middle school students. Chin. Ment. Health J..

[10] Zhang H., Wang Z., Liu Q., Yan C., Lei F., Wang R., Zhang J., Song G. (2022). Analysis of influencing factors of depressive symptoms among middle school students. Mod. Prev. Med..

[11] Zhang H., Chang K., Zhang F., Greenberger E., Chen C. (2011). Mental health problems and coping styles of urban and rural high school students in China. J. Community Psychol..

[12] Xu F., Huang Y. (2013). Advancements in adolescent depression symptoms research. Chin. J. Sch. Health.

[13] Yang J., Lü H., Xu N., Wang J., Ding X., Zhao D. (2022). Analysis of influencing factors of anxiety and depression in adolescents and evaluation of intervention effect of mindfulness-based cognitive therapy. J. Lanzhou Univ. (Med. Sci.).

[14] MacPhee A.R., Andrews J.J. (2006). Risk factors for depression in early adolescence. Adolescence.

[15] Watson D., Friend R. (1969). Measurement of social-evaluative anxiety. J. Consult. Clin. Psychol..

[16] Stein D.J., Lim C., Roest A.M., de Jonge P., Aguilar-Gaxiola S., Al-Hamzawi A., Alonso J., Benjet C., Bromet E.J., Bruffaerts R. (2017). The cross-national epidemiology of social anxiety disorder: Data from the World Mental Health Survey Initiative. BMC Med..

[17] Wang W., Xie X., Wang X., Lei L., Hu Q., Jiang S. (2019). Cyberbullying and depression among Chinese college students: A moderated mediation model of social anxiety and neuroticism. J. Affect. Disord..

[18] Soares F.C., Barros M., Bezerra J., Santos S.J., Machado L., Lima R.A. (2019). The synergic relationship of social anxiety, depressive symptoms and waist circumference in adolescents: Mediation analysis. J. Affect. Disord..

[19] Zhang C., Zhou Z. (2018). Passive Social Network Site Use, Social Anxiety, Rumination and Depression in Adolescents: A moderated mediating effect analysis. Chin. J. Clin. Psychol..

[20] Grant D.M., Judah M.R., Mills A.C., Lechner W.V., Davidson C.L., Wingate L.R. (2014). Rumination and Excessive Reassurance Seeking: Mediators of the Relationship Between Social Anxiety and Depression?. J. Psychopathol. Behav. Assess..

[21] Kessler R.C., Chiu W.T., Demler O., Merikangas K.R., Walters E.E. (2005). Prevalence, severity, and comorbidity of 12-month DSM-IV disorders in the National Comorbidity Survey Replication. Arch. Gen. Psychiatry.

[22] Cummings C.M., Caporino N.E., Kendall P.C. (2014). Comorbidity of anxiety and depression in children and adolescents: 20 years after. Psychol. Bull..

[23] Danneel S., Nelemans S., Spithoven A., Bastin M., Bijttebier P., Colpin H., Van Den Noortgate W., Van Leeuwen K., Verschueren K., Goossens L. (2019). Internalizing Problems in Adolescence: Linking Loneliness, Social Anxiety Symptoms, and Depressive Symptoms Over Time. J. Abnorm. Child. Psychol..

[24] Van Zalk N., Van Zalk M. (2019). Longitudinal Links Between Adolescent Social Anxiety and Depressive Symptoms: Testing the Mediational Effects of Cybervictimization. Child. Psychiatry Hum. Dev..

[25] Kobezak H.M., Gibb B.E. (2020). Prospective associations between social anxiety and depression in youth: The moderating role of maternal major depressive disorder. J. Adolesc..

[26] Radetzki P.A., Wrath A.J., Le T., Adams G.C. (2021). Alexithymia is a mediating factor in the relationship between adult attachment and severity of depression and social anxiety. J. Affect. Disord..

[27] Sun M., Liu K. (2018). The mediating role of loneliness in the relationship between social anxiety and depression symptoms in middle school students. Chin. J. Health Stat..

[28] Wu X., Huang L., He X., Tang H., Pu W. (2015). Social anxiety, aggression and depression: The mediating of regulatory emotional self-efficacy. Chin. J. Clin. Psychol..

[29] Lima R.A., de Barros M., Dos S.M., Machado L., Bezerra J., Soares F.C. (2020). The synergic relationship between social anxiety, depressive symptoms, poor sleep quality and body fatness in adolescents. J. Affect. Disord..

[30] Alcaraz-Ibanez M., Sicilia A., Paterna A. (2019). Exploring the differentiated relationship between appearance and fitness-related social anxiety and the risk of eating disorders and depression in young adults. Scand. J. Psychol..

[31] Jacobson N.C., Newman M.G. (2014). Avoidance mediates the relationship between anxiety and depression over a decade later. J. Anxiety Disord..

[32] Vaananen J.M., Isomaa R., Kaltiala-Heino R., Frojd S., Helminen M., Marttunen M. (2014). Decrease in self-esteem mediates the association between symptoms of social phobia and depression in middle adolescence in a sex-specific manner: A 2-year follow-up of a prospective population cohort study. BMC Psychiatry.

[33] Starr L.R., Hammen C., Connolly N.P., Brennan P.A. (2014). Does relational dysfunction mediate the association between anxiety disorders and later depression? Testing an interpersonal model of comorbidity. Depress. Anxiety.

[34] Wood J.V. (1989). Theory and Research Concerning Social Comparisons of Personal Attributes. Psychol. Bull..

[35] Swallow S.R., Kuiper N.A. (1988). Social comparison and negative self-evaluations: An application to depression. Clin. Psychol. Rev..

[36] Festinger L. (1954). A theory of social comparison processes. Hum. Relat..

[37] Xing S., Yu G. (2005). A review on research of social comparison. Adv. Psychol. Sci..

[38] Feinstein B.A., Hershenberg R., Bhatia V., Latack J.A., Meuwly N., Davila J., Kaufman J.C. (2013). Negative Social Comparison on Facebook and Depressive Symptoms: Rumination as a Mechanism. Psychol. Pop. Media Cult..

[39] Sloman L., Gilbert P., Hasey G. (2003). Evolved mechanisms in depression: The role and interaction of attachment and social rank in depression. J. Affect. Disord..

[40] Hwang H.S. (2019). Why Social Comparison on Instagram Matters: Its impact on Depression. KSII Trans. Internet Inf. Syst..

[41] Ding Q., Zhang Y., Zhang C., Du H., Zhou Z. (2016). Relationship between social network site use and depression in adolescents: Multiple mediating effect of social comparison and self-concept clarity. Chin. J. Clin. Psychol..

[42] Thwaites R., Dagnan D. (2004). Moderating variables in the relationship between social comparison and depression: An evolutionary perspective. Psychol. Psychother..

[43] Van Deurzen I., Van Ingen E., Van Oorschot W.J.H. (2015). Income Inequality and Depression: The Role of Social Comparisons and Coping Resources. Eur. Sociol. Rev..

[44] Osafo H.H., Wood A.M., Brown G.D., Dunn G. (2015). Why does Income Relate to Depressive Symptoms? Testing the Income Rank Hypothesis Longitudinally. Soc. Indic. Res..

[45] Lestari M.A., Solekhah T.A. (2022). The Relationship Between Social Comparison with Social Anxiety in Youth Users Instagram in Jakarta. J. Impresi Indones..

[46] Xue S. (2020). The Research on Current Situation and Relationship Among Interaction Anxiety, Upward Social Comparison, Self-Esteem and Body Image Satisfaction. Master’s Thesis.

[47] Cody M.W., Teachman B.A. (2010). Post-event processing and memory bias for performance feedback in social anxiety. J. Anxiety Disord..

[48] Zaffar W., Arshad T. (2020). The relationship between social comparison and submissive behaviors in people with social anxiety: Paranoid social cognition as the mediator. Psych. J..

[49] Zhang Y., Zhou Z., Zhu X., Lian S. (2017). The impact of social network site use on adolescents’ happiness: A moderated mediation model. Stud. Psychol. Behav..

[50] Turner R.J., Turner J.B., Hale W.B., Johnson R.J., Turner R.J., Link B.G. (2014). Social Relationships and Social Support. Sociology of Mental Health: Selected Topics from Forty Years 1970s–2010s.

[51] Kahn R.L., Antonucci T.C., Baltes P.B., Grim O.G. (1980). Convoys over the life course: Attachment, roles, and social support. Life Span Development and Behavior.

[52] Coyne J.C., Whiffen V.E. (1995). Issues in personality as diathesis for depression: The case of sociotropy-dependency and autonomy-self-criticism. Psychol. Bull..

[53] Kang Y., Ha J., Ham G., Lee E., Jo H. (2022). A structural equatin model of the relationships between social-emotional competence, social support, depression, and aggression in early adolescents in South Korea. Child. Youth Serv. Rev..

[54] Cao T., Liu Y. (2017). The relationship between social support, hope, and depression in middle school students in Beijing and Harbin. Chin. J. Sch. Health.

[55] Rueger S.Y., Malecki C.K., Pyun Y., Aycock C., Coyle S. (2016). A meta-analytic review of the association between perceived social support and depression in childhood and adolescence. Psychol. Bull..

[56] Höltge J., Theron L., Ungar M. (2022). A multisystemic perspective on the temporal interplay between adolescent depression and resilience-supporting individual and social resources. J. Affect. Disord..

[57] Auerbach R.P., Bigda-Peyton J.S., Eberhart N.K., Webb C.A., Ho M.H. (2011). Conceptualizing the prospective relationship between social support, stress, and depressive symptoms among adolescents. J. Abnorm. Child. Psychol..

[58] Huurre T., Eerola M., Rahkonen O., Aro H. (2007). Does social support affect the relationship between socioeconomic status and depression? A longitudinal study from adolescence to adulthood. J. Affect. Disord..

[59] Li M., Ren Y., Sun H. (2020). The relationship between social anxiety, perceived social support, and hope among rural left-behind children. Chin. J. Sch. Health.

[60] Leeves S., Banerjee R. (2014). Childhood social anxiety and social support-seeking: Distinctive links with perceived support from teachers. Eur. J. Psychol. Educ..

[61] Weymouth B.B., Fosco G.M., Mak H.W., Mayfield K., LoBraico E.J., Feinberg M.E. (2019). Implications of interparental conflict for adolescents’ peer relationships: A longitudinal pathway through threat appraisals and social anxiety symptoms. Dev. Psychol..

[62] Gallagher M., Prinstein M.J., Simon V., Spirito A. (2014). Social anxiety symptoms and suicidal ideation in a clinical sample of early adolescents: Examining loneliness and social support as longitudinal mediators. J. Abnorm. Child Psychol..

[63] Xiao R., Wu W., Zhang W. (2007). The reliability and validity of the Chinese version of Social Phobia Inventory. West Chin. Med. J..

[64] Messer S.C., Angold A., Costello E.J., Loeber R., VanKammen W., Loeber M.S. (1995). Development of a short questionnaire for use in epidemiological studies of depression in children and adolescents: Factor composition and structure across development. Int. J. Methods Psychiatr. Res..

[65] Cheng P., Cao F., Su L. (2009). Reliability and Validity of the Short Mood and Feelings Questionaire in Chinese Adolescents. Chin. Ment. Health J..

[66] Connor K.M., Davidson J.R., Churchill L.E., Sherwood A., Foa E., Weisler R.H. (2000). Psychometric properties of the Social Phobia Inventory (SPIN): New self-rating scale. Br. J. Psychiatry.

[67] Liu X. (2023). Relationship Between Parenting Styles and Adolescents’ Depression: Serial Mediating Effects of Emotional Regulation and Social Anxieties. J. Yichun Univ..

[68] Gibbons F.X., Buunk B.P. (1999). Individual differences in social comparison: Development of a scale of social comparison orientation. J. Pers. Soc. Psychol..

[69] Wang M., Wang L., Shi J. (2006). Reliability and validation of the Social Comparison Orientation Scale. Chin. Ment. Health J..

[70] Zhao X., Li L., Li L. (2024). Relationship between Social Comparison and Online Compulsive Buying in Vocational School Students: A Moderated Mediation Model. Chin. J. Clin. Psychol..

[71] Zimet G.D., Dahlem N.W., Zimet S.G., Farley G.K. (1988). The Multidimensional Scale of Perceived Social Support. J. Pers. Assess..

[72] Yang X., Han X. (2021). Reliability and validity of the Chinese version of the Multidimensional Scale of Perceived Social Support in primary and secondary school students. Chin. J. Clin. Psychol..

[73] Uriarte-Gaspari L., Acuna A., Morales S., Fernandez-Theoduloz G., Paz V., Perez A., Cabana A., Gradin V.B. (2022). Who do I want in my team: Social avoidance of high qualified partners in depression and social anxiety. J. Affect. Disord. Rep..

[74] Mitchell M.A., Schmidt N.B. (2014). An experimental manipulation of social comparison in social anxiety. Cogn. Behav. Ther..

[75] Butzer B., Kuiper N.A. (2006). Relationships between the frequency of social comparisons and self-concept clarity, intolerance of uncertainty, anxiety, and depression. Pers. Individ. Differ..

[76] Sun Q., Liu Y. (2020). The Effect of social comparison on college students’ depression: A moderated mediating effect. Psychol. Explor..

[77] Wu Y., Wu L., Niu G., Chen Z., Wang L. (2020). The influence of WeChat moments use on undergraduates’ depression: The effects of negative social comparison and self-concept clarity. Psychol. Dev. Educ..

[78] Lian S., Sun X., Niu G., Zhou Z. (2017). Upward social comparison on SNS and depression: A moderated mediation model and gender difference. Acta Psychol. Sin..

[79] Niu G., Sun X., Zhou Z., Kong F., Tian Y. (2016). The impact of social network site (Qzone) on adolescents’ depression: The serial mediation of upward social comparison and self-esteem. Acta Psychol. Sin..

[80] Joormann J., Arditte K., Gotlib I.H., Hammen C.L. (2014). Cognitive aspects of depression. Handbook of Depression.

[81] Gilbert P. (2000). The relationship of shame, social anxiety and depression: The role of the evaluation of social rank. Clin. Psychol. Psychother..

[82] Ren Y., Han P., Gao F., Han L. (2017). Shyness and material value: Multiple mediating of self-control and social comparison orientation. Chin. J. Clin. Psychol..

[83] Baldwin M., Mussweiler T. (2018). The culture of social comparison. Proc. Natl. Acad. Sci. USA.

[84] Kemmelmeier M., Oyserman D. (2001). The ups and downs of thinking about a successful other: Self-construals and the consequences of social comparisons. Eur. J. Soc. Psychol..

[85] Chung T., Mallery P. (1999). Social comparison, individualism-collectivism, and self-esteem in China and the United States. Curr. Psychol..

[86] Cohen A.B., Wu M.S., Miller J. (2016). Religion and Culture: Individualism and Collectivism in the East and West. J. Cross Cult. Psychol..

[87] Essau C.A., Ishikawa S., Sasagawa S., Sato H., Okajima I., Otsui K., Georgiou G.A., O’Callaghan J., Michie F. (2011). Anxiety symptoms among adolescents in Japan and England: Their relationship with self-construals and social support. Depress. Anxiety.

[88] Burt R.S. (2004). Structural Holes and Good Ideas. Am. J. Sociol..

[89] Coleman J.S. (1990). Forms of Social Capital. Foundations of Social Theory.

[90] Wellman B., Wortley S. (1989). Brothers’ Keepers: Situating Kinship Relations in Broader Networks of Social Support. Sociol. Perspect..

[91] Lin N., Marsden P.V., Lin N. (1982). Social Resources and Instrumental Action. Social Structure and Network Analysis.

[92] Granovetter M.S. (1974). Getting a Job. A Study of Contacts and Careers.

[93] Chang C., Yuan R., Chen J. (2018). Social support and depression among Chinese adolescents: The mediating roles of self-esteem and self-efficacy. Child. Youth Serv. Rev..

[94] McPherson K.E., Kerr S., McGee E., Morgan A., Cheater F.M., McLean J., Egan J. (2014). The association between social capital and mental health and behavioural problems in children and adolescents: An integrative systematic review. BMC Psychol..

[95] O’Connor M., Hawkins M.T., Toumbourou J.W., Sanson A., Letcher P., Olsson C.A. (2011). The relationship between social capital and depression during the transition to adulthood. Aust. J. Psychol..

[96] Li Y. (2021). A meta-analysis of the impact of social support on depression in junior high school students. J. Shanghai Educ. Res..

[97] Tian L., Chen G., Wang S., Liu H., Zhang W. (2012). Effects of parental support and friendship support on loneliness and depression during early and middle adolescence. Acta Psychol. Sin..

[98] Cole D.A., Preacher K.J. (2014). Manifest Variable Path Analysis: Potentially Serious and Misleading Consequences due to Uncorrected Measurement Error. Psychol. Methods.

